# Regional Differences in Tight Junction Protein Expression in the Blood–DRG Barrier and Their Alterations after Nerve Traumatic Injury in Rats

**DOI:** 10.3390/ijms21010270

**Published:** 2019-12-31

**Authors:** Thomas J. Lux, Xiawei Hu, Adel Ben-Kraiem, Robert Blum, Jeremy Tsung-Chieh Chen, Heike L. Rittner

**Affiliations:** 1 Department of Anaesthesiology, University Hospital of Wuerzburg, 97074 Wuerzburg, Germany; thomas.lux@stud-mail.uni-wuerzburg.de (T.J.L.); xiawei_hu@163.com (X.H.); Ben_A@ukw.de (A.B.-K.); 2 Institute of Clinical Neurobiology, University Hospital of Wuerzburg, 94074 Wuerzburg, Germany; Blum_R@ukw.de

**Keywords:** tight junction, claudin-5, neuropathic pain, nerve injury, dorsal root ganglion

## Abstract

The nervous system is shielded by special barriers. Nerve injury results in blood–nerve barrier breakdown with downregulation of certain tight junction proteins accompanying the painful neuropathic phenotype. The dorsal root ganglion (DRG) consists of a neuron-rich region (NRR, somata of somatosensory and nociceptive neurons) and a fibre-rich region (FRR), and their putative epi-/perineurium (EPN). Here, we analysed blood–DRG barrier (BDB) properties in these physiologically distinct regions in Wistar rats after chronic constriction injury (CCI). *Cldn5*, *Cldn12*, and *Tjp1* (rats) mRNA were downregulated 1 week after traumatic nerve injury. Claudin-1 immunoreactivity (IR) found in the EPN, claudin-19-IR in the FRR, and ZO-1-IR in FRR-EPN were unaltered after CCI. However, laser-assisted, vessel specific qPCR, and IR studies confirmed a significant loss of claudin-5 in the NRR. The NRR was three-times more permeable compared to the FRR for high and low molecular weight markers. NRR permeability was not further increased 1-week after CCI, but significantly more CD68^+^ macrophages had migrated into the NRR. In summary, NRR and FRR are different in naïve rats. Short-term traumatic nerve injury leaves the already highly permeable BDB in the NRR unaltered for small and large molecules. Claudin-5 is downregulated in the NRR. This could facilitate macrophage invasion, and thereby neuronal sensitisation and hyperalgesia. Targeting the stabilisation of claudin-5 in microvessels and the BDB barrier could be a future approach for neuropathic pain therapy.

## 1. Introduction

Neuropathic pain is defined as a “pain caused by a lesion or disease of the somatosensory nervous system”. The etiologies range from metabolic conditions like diabetes mellitus, autoimmune disorders, infectious diseases and chemotherapy-induced neuropathies to traumatic nerve injury and postoperative conditions. Overall, 7–10% of the general population are affected, and their quality of life is significantly lowered [1]. The heterogeneity of etiologies leading to neuropathic pain indicates that a multitude of not yet understood factors participate in the pathogenesis of neuropathic pain [2]. A causative therapy is currently often impossible [3]. While conventional analgesics, e.g., cyclooxygenase inhibitors such as ibuprofen, have little effect on neuropathic pain, symptomatic treatments include antidepressants, antiepileptics, and opioids, but the efficacy of these drugs is moderate [2]. Attempts to develop pathophysiology-oriented drugs have not delivered the desired results at this point. A better understanding of the mechanisms behind neuropathic phenotypes, therefore, is necessary to find new diagnostic and therapeutic approaches.

Several distinct animal models are used for research in neuropathy. Models are separated into peripheral nerve injury, central pain, drug-induced neuropathy, and disease-induced approaches [4]. Since the introduction of the chronic constriction injury (CCI) model for traumatic neuropathy in 1988 [1], it has been commonly used to understand the pathophysiology of mononeuropathies. Animals are subjected to four ligations of the sciatic nerve, which cause focal ischemia, intraneural edema, and Wallerian degeneration, and subsequently, regeneration. This results in a behavioral phenotype with signs of spontaneous pain and thermal and mechanical hypersensitivity [2]. Hypersensitivity reaches its maximum after 7 d [5], but nociceptive thresholds return to normal within the next 3–6 weeks depending on the noxious stimulus [6]. The local (inflammatory) reaction caused by cell damage at the ligature site does not explain the phenotype after CCI sufficiently. Changes in the dorsal root ganglion (DRG), such as infiltration of macrophages (ED1 for CD68 in rats) and CD8+ lymphocytes have been reported [7]. Furthermore, sprouting of sympathetic axons into the DRG after CCI has been observed [8]. These findings are thought to participate in the pathogenesis of traumatic mononeuropathies. Only about 30% of the neurons of the L4/5 DRGs are afferences from the sciatic nerve. Since other areas of the L4/5 dermatome show few to no changes after CCI, a combination of neuronal and environmental changes must be elementary. Electrophysiological and histopathological studies identified reduced nerve conduction velocity and axonal damage, as correlates of A-fiber damage and sensitization of C-fibers, as probable causes for this phenotype. 

Even slight environmental changes disrupt physiological function of neural tissues. Therefore, the entry points to the nervous system are protected. Highly specialized barriers guard myelinated fibers (myelin barrier, MB), the peripheral nerve (blood–nerve barrier), the DRG (blood–DRG barrier, BDB), the spinal cord (blood–spinal-cord barrier), and the brain (blood–brain barrier). The names originate from the protected region, and each of them can be subdivided into the different tissue interfaces, e.g., the endothelial vessels or the perineurium, each fulfilling different physiological demands. Key features for this sealing function are tight junction proteins. The specific tight junction protein composition determines the barriers characteristics. While many of these characteristics are similar in the blood–nerve barrier and blood–brain barrier, distinct features, such as absence of several neurotransmitter transporters and lack of astrocytes in the periphery, qualify the blood–nerve barrier as a specialized, unique structure [9]. 

After axonal injury or acute demyelination, extravasation of blood-borne molecules such as albumin and intraneural edema as correlates of blood–nerve barrier leakage can be observed [10]. Studies of the blood–nerve barrier in nerve injury models have revealed downregulation of *Cldn1*, *Cldn5*, *Ocln*, *Tjp1* (ZO-1) and *Jam3* (JAMC) mRNA in the sciatic nerve [10,11,12,13,14] as well as *Tjp1* (ZO-1), *Cldn5* and *Ocln* in the spinal cord of rats [15,16]. Similar studies observed reductions of *Cldn1* and *Tjp1* (ZO-1) mRNA and immunoreactivity (IR) in the sciatic nerves of mice [13]. Endothelial cells of the blood–nerve barrier are disrupted with increased permeability as soon as 6 h after CCI surgery, while the neuropathic phenotype develops over days [9]. So, barrier disruption occurs early after nerve injury even before hypersensitivity. 

Surprisingly, the BDB has barely been studied before. Regions in the DRG can be divided: Somata of primary sensory neurons reside in DRGs (neuron rich region (NRR)) in contrast to fiber rich regions (FRR). It is known that the BDB is considerably more permeable and contains a higher density of capillaries [17,18,19]. Claudin-5 IR was detected in the NRR, while claudin-1 and occludin were found in the FRR [19]. However, no quantitative data of either protein or mRNA in all areas, including epi-/perineurium (EPN), are currently available in naïve animals or after neuropathy. Furthermore, whole tissue analysis can be insensitive to small changes of specific barriers and novel techniques, making selective analysis necessary. 

In this study, we wanted to fully characterize the BDB and its alteration in neuropathy. To this end, we defined four regions in the DRG: the neuron-rich and the fiber-rich regions (NRR, FRR), and their putative epi-/perineurial regions (NRR-EPN, FRR-EPN). We used these regions and region-selective techniques to analyze typical tight junction proteins known from the blood nerve and myelin barrier in control rats and in neuropathy and evaluated functional properties of the BDB. Tight junction proteins, including claudin-1, were detected in the nerve perineurium, claudin-5 was found in endoneurial vessels, ZO-1 was ubiquitously detected in the nerve, and claudin-12 and 19 were present in Schwann cells. 

## 2. Results

### 2.1. Claudin-1, Claudin-19, and ZO-1 Immunoreactivity Is Tissue Specific in Rat DRGs

To characterize the BDB and its molecular structure, we quantified the immunoreactivity (IR) in the DRG, considering its different regions. After separating the DRGs distinct areas, claudin-1, claudin-5, claudin-12, claudin-19, and ZO-1, IR was semi-quantified and compared between the NRR, FRR, and the putative EPN of DRGs after CCI. 

**Figure 1:**
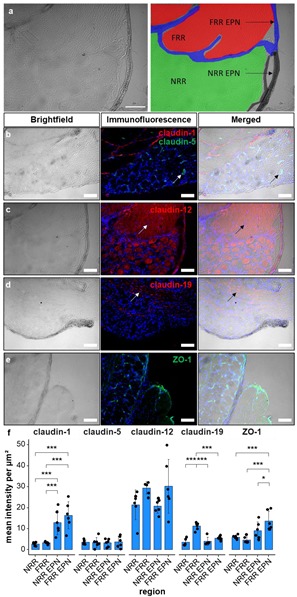
Claudin-1 immunoreactivity (IR) and ZO-1 IR are preferentially found in epi-/perineurium in rats’ dorsal root ganglia (DRGs), while claudin-19 IR is most abundant in the fiber-rich region. Classification into neuron rich region (NRR, green), NRR epi-/perineurium (EPN, black), fiber rich region (FRR, red), and FRR-EPN (blue) is shown in (**a**). Control DRG sections (CL) from Wistar rats were immunostained. IRs of claudin-1, claudin-5, and ZO-1 were quantified and compared between NRR and FRR, together with their putative EPN. Representative stainings for claudin-1 (red), claudin-5 (green) (**b**), claudin-12 (**c**), claudin-19 (**d**), and ZO-1 (**e**) are shown. Arrows point to structures identified as vessels (**b**), Schwann cells (**c**), and paranodes of Schwann cells (**d**). Quantification of the signal intensity in the specified areas (**f**). (Scale bars = 100 µm; *n* = 5 or 6; claudin-1: NRR versus NRR-EPN, NRR versus FRR-EPN, FRR versus NRR-EPN, and FRR versus FRR-EPN: *p* < 0.0001; ZO-1: FRR-EPN versus NRR: *p* = 0.0003; FRR-EPN versus NRR-EPN: *p* = 0.0138; FRR-EPN versus NRR: *p* = 0.00018. No normal distribution: ZO-1 FRR. No variance homogeneity: claudin-1. Two-way ANOVA, Tukey HSD. * *p* < 0.05, *** *p* < 0.001).

While claudin-1 IR was up to five times higher in the EPN as in the inner regions of the DRG (Figure 1b,f), claudin-19 IR was highest in the FRR (Figure 1d,f) and ZO-1 IR was highest in the FRR-EPN (Figure 1e,f). Claudin-5 and claudin-12 expressions were not region specific. The mean intensity of claudin-5 IR was low, but areas with strong signals, mostly associated with claudin-1 signals, were observed (Figure 3). In brightfield images, these areas resembled vessels. This was also seen for ZO-1-IR, which was expressed in the structures resembling capillaries and in the EPN. Claudin-12 was not only found in putative Schwann cell structures, but neurons as well. In contrast, claudin-19-IR was detected in typical paranodal structures of Schwann cells. 

### 2.2. Nocifensive Responsiveness after Nerve Injury

We next wanted to test whether neuropathy results in alterations of the BDB. To demonstrate the validity of the neuropathy CCI model, mechanical nociceptive thresholds were recorded for both rear limbs before and one week after CCI via the von-Frey test. 

**Figure 2:**
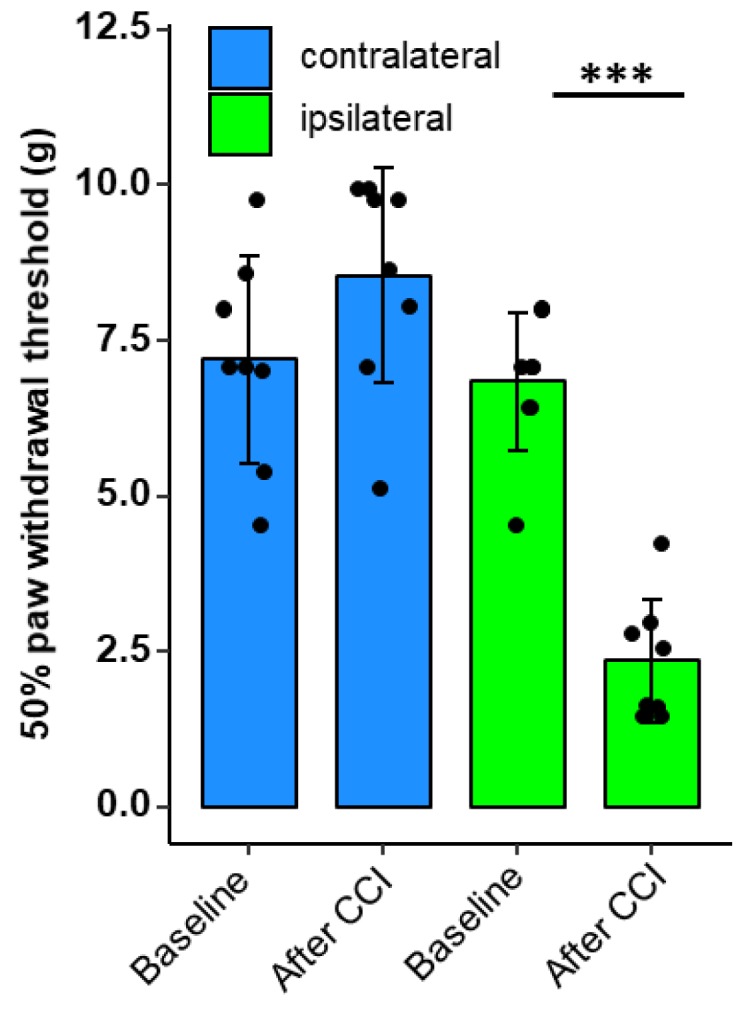
Mechanical hypersensitivity in rats after chronic constriction injury (CCI). Male Wistar rats underwent chronic constriction injury (CCI). Mechanical nociceptive thresholds were evaluated before and one week after CCI (*n* = 8, Von-Frey test; repeated measures ANOVA, *** *p* < 0.001).

While there was no change in the sensitivity in the contralateral paw, the limb with nerve injury exhibited a decrease of the mechanical withdrawal threshold to 34% of the previous baseline value (Figure 2), indicating mechanical hypersensitivity in neuropathy. 

### 2.3. Downregulation of Tight Junction Protein mRNA Expression after CCI in Rodents

**Figure 3:**
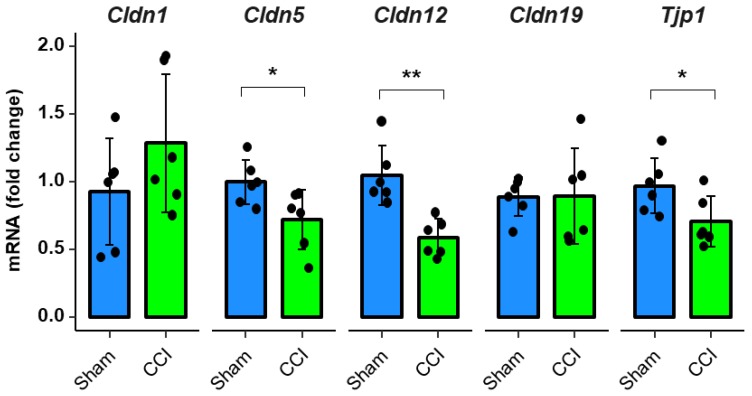
Tight junction protein mRNA expression in rat DRGs is downregulated one week after CCI. Wistar rats were treated with CCI. Relative mRNA expressions of *Cldn1*, *Cldn5*, *Cldn12*, *Cldn19*, and *Tjp1* (ZO-1) in comparison to sham operated animals were analyzed with qPCR after one week. (*n* = 6; *Cldn5*: *p* = 0.0338; *Cldn12* *p* = 0.0021, *Tjp1* (ZO-1) *p* = 0.0419. No variance homogeneity: *Cldn19* rats. Welch two-sample *t*-test, Wilcoxon rank sum test: * *p* < 0.05, ** *p* < 0.01).

For evaluation of tight junction protein expression patterns during CCI, we analyzed the mRNA levels of *Cldn1*, *Cldn5*, *Cldn12*, *Cldn19,* and *Tjp1* (ZO-1) in rats’ whole DRGs. The expression levels of *Cldn5*, *Cldn12*, and *Tjp1* (ZO-1) mRNA were reduced by 28%, 45% and 26%, respectively after CCI compared to sham (Figure 3).

### 2.4. Claudin-5 Expression in Vessels Is Reduced Only in the NRR after CCI

**Figure 4:**
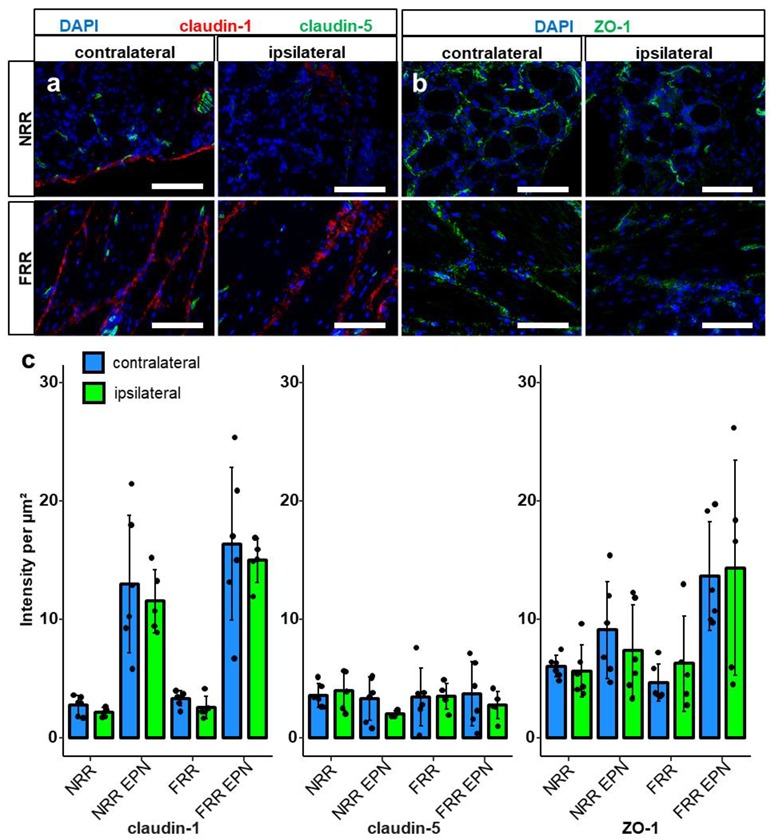
Claudin-1 and ZO-1 IR are unchanged after CCI. DRG sections from Wistar rats with 7 d CCI and naïve controls were immunostained. IRs of claudin-1, claudin-5, and ZO-1 were quantified and compared between IL and CL DRGs after CCI in the neuron rich region (NRR) and fiber rich region (FRR), and their putative EPN are presented as light intensity per µm^2^. Representative sections of claudin-1 (red) and claudin-5 (green) (**a**), and ZO-1 (green) (**b**) are shown. Quantification of the signal intensity in the specified areas: significant differences between regions were not indicated, as they are already analyzed in Figure 1 (**c**). (Scale bars = 100 µm. *n* = 5 or 6; claudin-1: NRR versus NRR-EPN, NRR versus FRR-EPN, FRR versus NRR-EPN, and FRR versus FRR-EPN: *p* < 0.0001; ZO-1: FRR-EPN versus NRR: *p* = 0.0003; FRR-EPN versus NRR-EPN: *p* = 0.0138; FRR-EPN versus NRR: *p* = 0.00018. No normal distribution: ZO-1 CL FRR. No variance homogeneity: claudin-1 CL. Two-way ANOVA, Tukey HSD).

**Figure 5:**
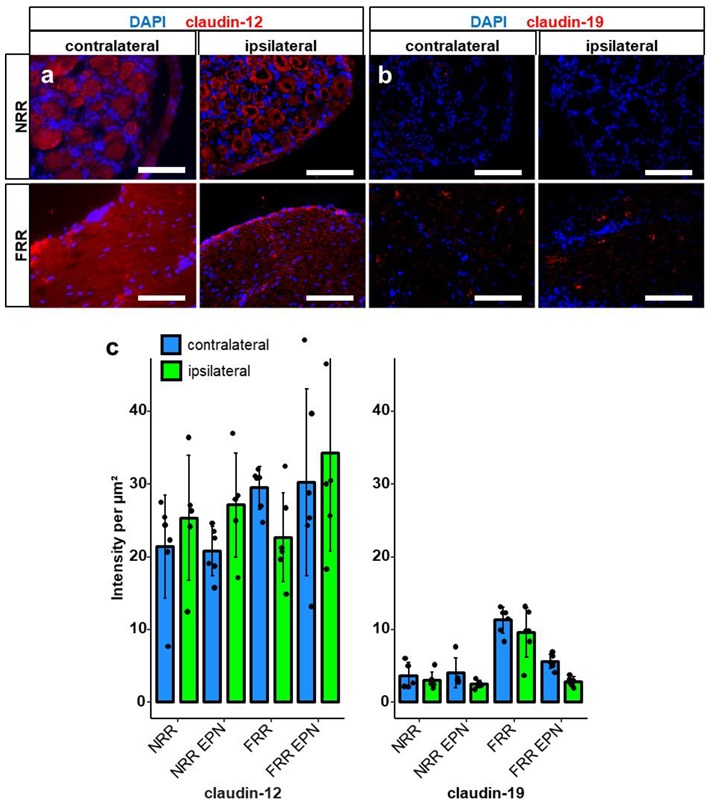
Claudin-19 and claudin-12-IR are unaltered after CCI. After CCI, DRG sections of Wistar rats were immunostained. IRs of claudin-12 and claudin-19 were quantified as light intensity per µm^2^. and compared between IL and CL DRGs after CCI in the NRR and FRR, and their putative EPNs. Representative sections of claudin-12 (red) (**a**) and claudin-19 (red) (**b**) are shown. Quantification of the signal intensity in the specified areas: significant differences between regions are not indicated, as they were already analyzed in Figure 1 (**c**). (Scale bars = 100 µm. *n* = 5 or 6; claudin-19: FRR versus NRR, FRR versus NRR-EPN, and FRR versus FRR-EPN: *p* < 0.0001. No normal distribution: claudin-19 CL NRR-EPN. Two-way ANOVA, Tukey HSD).

Comparison of the tight junction protein IR between naive and CCI rats in the distinct regions did not reveal significant differences (Figure 4 and Figure 5). Only a tendency was observed for claudin-12 in the FRR and claudin-19 for the FRR-EPN (*p* = 0.057). 

**Figure 6:**
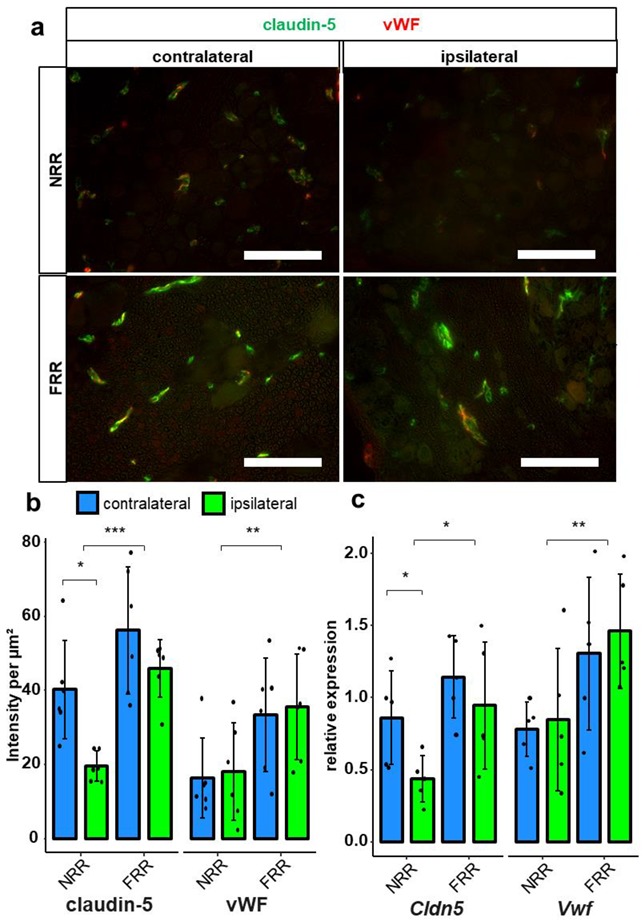
Claudin-5 IR within vessels and *Cldn5* mRNA in the NRR are reduced in rat DRGs. DRG sections from Wistar rats after CCI were immunostained. Quantification of claudin-5 and van Willebrand factor (vWF) IRs and comparison between IL and CL DRGs after CCI in the NRR and FRR as light intensity per µm^2^. Tissue samples from the NRR and vessels of the FRR were obtained using laser microdissection (LMD). Claudin-5 and vWF mRNA were quantified as relative expression (to GAPDH) via RT-qPCR. Representative sections of co-stainings with claudin-5 (green) and vWF (red) (**a**). Quantification of the claudin-5 and vWF IR (**b**) *n* = 6; claudin-5: FRR versus NRR, *p* = 0.000261; NRR: CL versus CCI, *p* = 0.02985; vWF: NRR versus FRR, *p* = 0.00515. No normal distribution: claudin-5 FRR CCI, vWF NRR CL. No variance homogeneity: claudin-5 FRR. Two-way ANOVA, Tukey HSD.) And quantification of mRNA expression (**c**) *n* = 5; *Cldn5*: NRR versus FRR, *p* = 0.0136; CL versus IL: *p* = 0.0448; *Vwf*: NRR versus FRR, *p* = 0.00838; two-way ANOVA, Tukey HSD) in the specified areas. (Scale bars = 100 µm. * *p* < 0.05, ** *p* < 0.01, *** *p* < 0.001.) All results are summarized in Table 1.

Since the method is optimized for homogenously expressed proteins and lacks sensitivity for proteins with clustered expression, e.g., in vessels, we analyzed claudin-5 using a different approach. We co-stained the tissue with von Willebrand Factor (vWF) to analyze capillaries in the NRR and FRR (Figure 6). Selective analysis of vessels revealed higher vWF IR in vessels of the FRR compared with the NRR, but no change after CCI. Analysis of claudin-5 within vessels could also show higher IR in the FRR than in the NRR area. Most importantly, we observed a reduction of claudin-5 signal in the vessels of the NRR after CCI. 

To verify these results on a mRNA level, we dissected capillaries from the FRR and the NRR by laser dissection in sham and CCI rats 1 d to reflect the mRNA alterations preceding protein expression. No changes were observed for *Vwf* and *Cldn5* mRNA in the FRR, but *Cldn5* was significantly downregulated in the NRR. 

### 2.5. Permeability of the DRG and Migration of Macrophages

**Figure 7:**
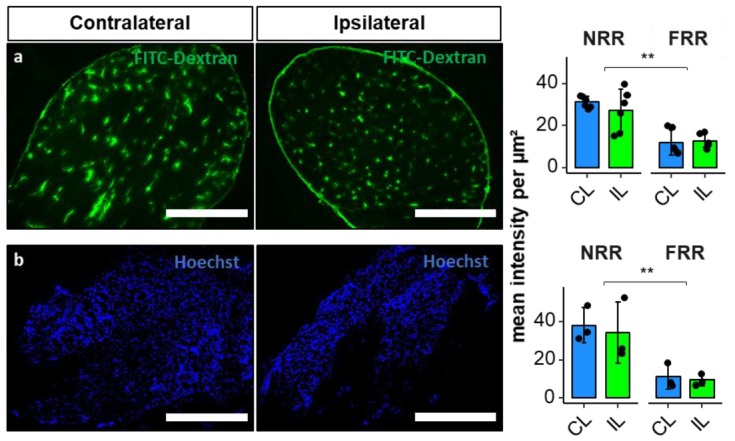
Blood–DRG barrier (BDB) permeability for large molecules in the NRR is higher compared to FRR of DRGs, but has no change after CCI. Wistar rats after 7 d CCI were perfused with FITC-dextran ((**a**), 70 kDa) and Hoechst reagent ((**b**), 562 Da). NRR and FRR of ipsilateral (IL) and contralateral (CL) DRG sections were analyzed. Representative images of the IL show comparable IR. Quantification of FITC-dextran (*n* = 6, NRR versus FRR: *p* < 0.001; two-way ANOVA, Tukey HSD) and Hoechst (*n* = 3, NRR versus FRR *p* = 0.002; two-way ANOVA, Tukey HSD) Immunofluorescence as intensity per µm^2^. (Scale bars = 100 µm; ** *p* < 0.01.)

To finally evaluate functional properties of the BDB, we established an assay for vessel permeability with FITC-dextran (70 kDa). After intravenous application, the dye accumulated in the perineurium and within vessels, diffusing a few micrometers into the tissue. Hence, the NRR was 2.35 times more permeable than the FRR; however, no difference after CCI was observed (Figure 7). In the next step, we analyzed the permeability for small molecules using Hoechst reagent (562 Da) after systemic administration in vivo. Hoechst reagent ubiquitously diffused into the tissue and then stained nuclei. We detected significantly higher (3.51 times) permeability in the NRR region compared to the FRR but no change after CCI. 

**Figure 8:**
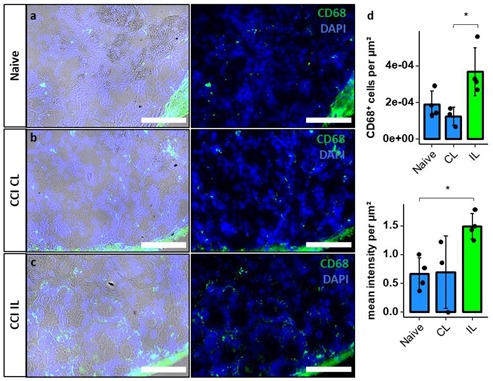
Increased macrophage invasion in the NRR after CCI. DRGs of naive (**a**) and contralateral (CL) (**b**) and ipsilateral (IL) (**c**) DRGs of Wistar rats 7d after CCI were harvested and stained using DAPI and anti-CD68 antibodies (left: immunofluorescence with brightfield; right: only immunofluorescence). In the NRR, CD68+ cells were counted manually, and signal intensity was measured. Both were quantified per µm^2^ (**d**). (*n* = 3 or 4; CD68+ cells per µm^2^: CL versus IL, *p* = 0.024; mean intensity per µm^2^: Naive versus IL, *p* = 0.038. Two-way ANOVA, Tukey HSD; scale bars = 100 µm; * *p* < 0.05).

CD68^+^ cells and CD68 immunoreactivity was quantified in the NRR in naive animals DRGs, and contralateral and ipsilateral DRGs after CCI to assess macrophage migration (Figure 8). CD68^+^ cells per µm^2^ were significantly increased in the IL DRGs after CCI compared to CL, while there was only a tendency (*p* = 0.065) compared to naive DRGs. The anti-CD68 immunoreactivity per µm^2^ was significantly higher in IL DRGs after CCI, compared to naive DRGs.

## 3. Discussion

In our study we further characterized the BDB and examined whether traumatic neuropathy not only results in blood–nerve barrier and blood spinal cord barrier breakdown but also affects the BDB. We revealed higher ZO-1 expression in the FRR EPN compared to the NRR and observed regional expression differences for claudin-1 (highest expression in NRR ENP and FRR EPN), claudin-5 (higher expression in the FRR compared to the NRR), and claudin-19 (highest expression in the FRR), as indicated in Table 1. After CCI, claudin-5 IR and *Cldn5* mRNAs were selectively reduced in the vessels of the NRR. The NRR containing somatosensory neurons was 2–3 times more permeable to low and high molecular weight dyes compared to the nerve fiber area with no further increase in permeability after CCI. However more CD68+ macrophages migrated into the NRR. 

While the blood–nerve barrier is focus of current literature, the BDB is, so far, almost neglected. As an imperative link between the peripheral nerve and the spinal cord, open questions should be discussed more thoroughly, as they could reveal important knowledge for understanding the peripheral sensory system. The BDB is very similar to the blood–nerve barrier regarding morphology and tight junction protein composition, but its NRR barrier is leaky in its physiological state compared to the blood–nerve barrier. In accordance with our data, intravenously injected Hoechst dye or FITC-dextran diffusion out of capillaries can already be observed in DRGs of naive rats, while dyes only accumulate in peripheral nerves after blood–nerve barrier breakdown [18,20]. A clinically relevant consequence of these characteristics is the accumulation of drugs in the DRG, which can be favorable in case of opioids or undesirable in case of chemotherapeutic drugs causing neuropathies. This hints towards a concept of the DRG being more than a simple relay-station, but being an important signal modulator sensitive to endogenous and exogenous stimuli [21]. In addition to molecules, cells can migrate into the DRG. Macrophages reside in the DRG and further migrate into the DRG to shape an immune response in neuropathy [22,23]. So, opening of the BDB could facilitate these pathophysiological processes.

We identified that claudin-1, a major sealing tight junction protein, is predominantly expressed in the EPN of both the NRR and the FRR, as they show the highest claudin-1 IRs. *Cldn1* KO is lethal due to dehydration, as claudin-1 is key component of the epidermal barrier in the stratum granulosum in mice [24]. Besides this and other vital functions, claudin-1 is physiologically expressed in the peripheral and central nervous system as part of the blood–nerve barrier and blood–brain barrier. Studies of peripheral nerves observed high expression of *Cldn1* in the perineurium [13,25,26], but also in Schmidt-Lantermann incisures, paranodal loops of myelinating Schwann cells, and the mesaxon [8,27]. Previous studies performed on rats [12,28] and mice [13] also described a reduction of *Cldn1* mRNA and protein levels in the sciatic nerve after nerve injury. Downregulation of *Cldn1* mRNA starts around 3 h after CCI, and is lowest after 7 d [12]. In the DRG, we determined no significant differences of *Clnd1* mRNA in the whole DRG and claudin-1-IR in the subregions between naïve and CCI groups. Therefore, the EPN around rat DRGs seems not to be affected by CCI, although it is possible that intraspecies differences exist for mice, and more importantly, humans, in this and other barrier proteins. 

Expression of claudin-12 by glial cells has been observed in the blood–brain barrier [29,30] and blood–nerve barrier [31]. We confirmed *Cldn12* mRNA expression in the DRG. High levels of claudn-12 IR were detected within the somata, and in or around the fibers of neurons. The role of claudin-12 during nerve injury has not been examined yet. In our study, *Cldn12* mRNA was downregulated after CCI in the DRG. Even though we could not observe lower claudin-12 IR, the signal morphology seems disturbed from a more homogenous to a clustered distribution after CCI. While the biological relevance of the signal in the soma is unclear, the distribution and the change of claudin-12 after CCI should certainly be investigated further.

Claudin-19 is expressed in myelinating glia cells in the PNS, but not the CNS [32]. Tight junction formation in myelinating cells is disrupted without claudin-19 [33]. *Cldn19* KO mice are fertile and vital, but show the phenotype of a peripheral neuropathy, causing mainly motor defects, but in 50%, nerve conduction deficits as well [32]. In our study, claudin-19 IR was clustered and highest in the FRR, and in the space between the neurons’ somata in the NRR. These clusters could very well resemble the paranodal regions, like those already described in the sciatic nerve [31]. In agreement previous findings of claudin-19 expression in vessels and the perineurium, there were low signals in the EPNs of both regions [32], although we have previously seen this in the nerve [34]. Neither *Cldn19* mRNA levels nor claudin-19 IR were altered in the DRG proximal to the CCI injury. 

ZO-1, an intracellular tight junction associated protein, forms complexes with claudin-1 and is required for correct arrangement of tight junctions [33]. *Tjp1*-KO is lethal, and disruption has been linked to neurological disorders [35]. ZO-1 is mostly expressed in the perineurium [31], vessels, and myelinated Schwann cells in the sciatic nerves of humans [31] and mice [36]. In our study, ZO-1 IR signal was detected predominantly in the EPN, like claudin-1’s IR, and in clustered regions in all areas. The ZO-1 clusters colocalized with claudin-5, and are, therefore, most likely vessels. *Tjp1* was downregulated in whole DRGs. In our IF study, we detected the highest levels of ZO-1 IR expression in the FRR-EPN but no alterations after CCI. These findings match with the existing data from studies in the mouse sciatic nerve [36].

Considered the most important tight junction protein of the brain barrier, *Cldn5* mRNA expression is >100-fold higher than other tight junction protein expression in endothelial cells [37]. While *Cldn5* KO mice are macroscopically vital, increased brain barrier permeation by molecules of <800 Da and death 10 d after birth were observed [38]. In the blood–nerve barrier, *Cldn5* is expressed in the endoneurial vessels, and the myelinating Schwann cells of humans [31] and rats [39]. In costainings, claudin-5 IR colocalized with vWF, indicating vessel-specific location. The density of claudin-5 IR within vessels was higher in the FRR than in the NRR. This was accompanied by lower permeability for FITC-dextran and Hoechst dye in the FRR. Therefore, we assume lower claudin-5 expression in vessels as a key-factor for leakiness of the NRR’s BDB under physiological conditions. A downregulation of *Cldn5* mRNA in the blood–nerve barrier has consistently been reported after nerve injury in rats [12,28]. In whole DRGs, we observed a *Cldn5* mRNA downregulation. Regional quantification of mean IR within vessels and regional *qPCR* also revealed claudin-5 downregulation after CCI, but only in the NRR. A lower *Cldn5* expression could significantly impact the function of the BDB, making it even more permeable for potentially harmful substances and immune cell invasion. Indeed, we confirmed increased invasion of macrophages in our model in the ipsilateral DRG after CCI, as shown previously in rats [22] and mice [23]. To evaluate possible BDB breakdown regarding permeability for small and large molecules, we performed a perfusion assay with FITC-dextran and Hoechst reagent as representative molecules with high and low molecular weights. We could detect higher permeation for both reagents in the NRR compared to the FRR but no change 1-week after CCI. Even though claudin-5 downregulation in brain vessels results in barrier breakdown accompanied by permeation of small molecules, there are several explanations as why we could not detect this in the DRG: Firstly, the permeability of the NRR in CL DRGs is high, so additional claudin-5 might have little additional impact. Secondly, our permeability assays were established in experimental setups where the baseline showed little to no signal, and therefore, were optimized to detect differences in “low-signal” scenarios. Therefore, high saturations probably caused lower sensitivity. Thirdly, possible compensatory mechanisms might prevent a further increase of BDB permeability, such that there simply is no change. Further studies investigating the BDB after, e.g., long-term CCI or impact of claudin-5 on immune cell migration could clarify these questions.

The structural peculiarities of the DRG are not very well understood: DRG neurons have no afferent synapses and the precise function of the pseudo-unipolar feature is still not known. The somata reside at the end of the T-stem; nevertheless, afferent spikes are connected to the cell bodies and many DRG neurons exhibit specialized membrane characteristics, necessary for the initiation of action potentials. The role of these membranes in signal transmission or generation is not known. Why is the DRG or at least the NRR not as well protected as the nerve? What are the implications? Indeed, not only capillaries but also satellite cells surrounding neurons are permeable. Marker molecules in the extracellular space can access the soma membrane of DRG neurons by diffusion independent of neuropathy [40]. It is, therefore, possible that DRGs carry out some as-yet unidentified chemosensory functions associated with the body’s internal milieu. Interference with this delicate homeostasis might lead to increased sensitivity in neuropathy [41]. Interestingly, selectively silencing ectopic activity in the DRG with low dose local anesthetics in neuropathy reduces hypersensitivity [42]. Hypersensitivity could have been triggered by proalgesic molecules bypassing the BDB in neuropathy. 

Both methods used, qPCR and IHC, provide specific insights into biological systems within their limitations: The great sensitivity of qPCR can prove even slight changes, but provides spatial resolution only if laser dissection is performed in addition. Furthermore, qPCR data need to be correlated with protein data to generate viable evidence. Antibody-based protein labelling on the other hand, provides an insight into expression and distribution in situ, if a sensitive and specific antibody is used. In our study mRNA data did not fully match our IHC results. While mRNA levels do not necessarily correlate with protein expression, methodological aspects also must be considered, especially for *Cldn5* expression. Whole DRG and LSM qPCR showed a significant difference between *Cldn5* mRNA levels after CCI. Our first method of semi quantification lacks sensitivity regarding proteins, which are not homogenously distributed within one specified region. Therefore, we opted to analyze claudin-5 IR in the vessel area in addition. In summary, tight junctions in the DRG can only be studied sufficiently if different tissues are analyzed separately. 

What are the clinical implications? One obvious problem in pain research is the lack of suitable biomarkers and objective measures of pain—especially possible malfunctions of the pain pathway in the primary afferent neuron in the pain pathway [43]. Thus, imaging of the DRG could fill this gap in certain painful conditions. Indeed, alterations of DRG volume have been noted in chemotherapy-induced neuropathy, and two genetic diseases, Fabry disease and neurofibromatosis [44,45,46,47,48]. Alterations in the permeability at the blood tissue interface have been observed in long-standing Fabry disease in the NRR, indicating a concomitant dysfunctional DRG perfusion [47,48]. In the case of Fabry disease, this observation could to be the consequence of glycolipid accumulation. Unfortunately, pain intensity was not examined in the study. Also, it is not known so far, whether other types of neuropathic pain, including nerve injury or metabolic diseases, also have alterations in the BDB, and specifically, an increased leakage, as observed in the nerve [49]. Nevertheless, magnetic resonance imaging and BDB permeability could be very early in vivo markers for involvement of the peripheral nervous system. Secondly, if BDB breakdown is clinically relevant and contributes to pain generation then stabilization of the BDB (e.g., the wnt [34] and hedgehog pathway [12], as shown for the blood–nerve barrier) could also improve pain relief. Claudin-5 is regulated by steroids [50]. So, one might speculate that the epidural steroids used in the clinic could also improve BDB barrier sealing. Thirdly, the leaky BDB—already under baseline conditions—could facilitate targeting of the ectopic firing from the DRG for pain treatment, e.g., via dilute local anesthetics [42,51]. 

## 4. Materials and Methods 

### 4.1. Animals

Twenty 8–12 weeks old male rats Wistar rats (Janvier) were kept in cages of six in a 12 h light cycle with water and food ad libitum. All animal protocols were approved by the local authorities (Regierung Unterfranken, RUF55.2.2-2532-2-612-16, 18 April 2018) and were in accordance with the ARRIVE guidelines.

### 4.2. Chronic Constriction Injury

Rats were randomized to the surgery and sham control groups. Surgery of the animals was performed under deep isoflurane anesthesia (1.8 vol%, fiO_2_). Adequate anesthesia was assumed when paw withdrawal was absent. After skin incision and exposition of the sciatic nerve by blunt preparation, four loose silk ligatures (Perma Silk 6.0, Ethicon Inc., Somerville, NJ, USA) were used in rats with approximately 1 mm spacing in between [10,52]. After loosely tightening the ligatures, the skin was stitched (Prolene 5.0, Ethicon Inc., Somerville, NJ, USA). For sham operations, the same procedure was performed without applying the silk ligatures. All procedures were performed unilaterally, and the following experiments used the IL DRGs. As controls we used sham surgery (qPCR) or CL DRGs.

### 4.3. Behavioural Tests

Mechanical nociceptive thresholds were tested with the von-Frey test [53]. Rats were positioned on a wire mesh. Filaments were applied in ascending order. The initially used hair value was 1 g. In general, the filaments were applied to the plantar surface of the ipsilateral and contralateral hind paw and were held for 1–3 s until the filaments were bent to an angle of 45°. Each limb was tested three times. We determined the withdrawal threshold of the hind-limbs to a mechanical stimulus by using 50% paw withdrawal threshold (PWT) method. 

### 4.4. Reverse Transcription qPCR (RT-qPCR)

Quantification of mRNA of whole DRGs was performed using qPCR. RNA was first extracted from the samples using TRIzol™ Reagent (Invitrogen, Carlsbad, CA, USA); then, reverse transcribed to cDNA using the High capacity cDNA Reverse Transcriptase Kit (Applied Biosystems, Foster City, CA, USA) according to the manufacturer’s instructions. For RT-qPCR, the PowerUpTM SYBR Green Master Mix was used following the manufacturer’s protocol with primers for *Cldn1*, *Cldn5*, *Cldn12*, *Cldn19*, and *Tjp1* (ZO-1) (Table 2).

Total RNA from capillaries and neuron rich areas from rats DRGs was extracted using the RNeasy^®^ Micro Kit (Qiagen, Venlo, The Netherlands). Total RNA (1 µg) was transcribed to cDNA using the high-capacity cDNA kit (Applied Biosystems, Foster City, CA, USA) following the manufacturer’s instructions. *Gapdh* and *18S* were used as reference genes for quantification. Analysis via qPCR was performed with the following primers with the Taqman method: *Cldn5* (Rn01753146_s1, Thermo Scientific, Waltham, MA, USA) and *Vwf* (Rn01492158_m1, Thermo Scientific, Waltham, MA, USA). qPCR analysis was carried out using the StepOnePlus Real-Time PCR System (Applied Biosystems, Foster City, CA, USA) with the following program: 95 °C for 20 s followed by 45 cycles at 95 °C for 1 s and 60 °C for 20 s. Samples were analyzed as triplicates. Relative quantification of mRNA was calculated using the 2^−ΔΔCT^ method, in which CT represents the threshold cycle value. 

### 4.5. Immunofluorescence and Microscopy

After euthanizing the rats, the DRGs were harvested, embedded in Tissue Tek O.C.T. Compound (Sakura Finetek Europe B.V., AV Alphen aan den Rijn, The Netherlands), and snap frozen in liquid nitrogen. The samples were stored at −20 °C until further processing. Cryosections of 10 µm thickness were cut at −20 °C in a cryostat (Leica Biosystems CM3050 S Research Cryostat, Leica Biosystems Nussloch GmbH, Nussloch, Germany) and the slides were stored at −20 °C.

For fixation, the slides were immersed in 4% paraformaldehyde (Sigma Aldrich, St. Louis, MO, USA) in PBS (Sigma Aldrich, St. Louis, MO, USA). The fixed samples were blocked with 10% donkey serum in 0.3% Triton X-100 (Sigma-Aldrich, St. Louis, MO, USA) in PBS for 1 h at room temperature. After blocking, slides were incubated with the putative primary antibody in 10% donkey serum in PBS for 16 h at 4 °C: rabbit claudin-1 antibody (1:100, Invitrogen, Waltham, MA, USA, number 51-9000), mouse claudin-5-Alexa488 conjugate antibody (1:200, Invitrogen, number 352588), claudin-12 (1:100, IBL, number 18801), rabbit claudin-19 (1:100, gift from Hou, St. Louis, USA [54]), mouse ZO-1-Alexa488 conjugate antibody (1:200, Invitrogen, number 339188), rabbit vWF antibody (1:100, Dako, Santa Clara, CA, USA, A0082), and mouse anti rat CD68 antibody (1:100; Bio-rad, Hercules, CA, USA, MCA341R). Claudin-1 and claudin-5 or vWF and claudin-5 antibodies were applied together as a co-stain. Primary antibody incubation was followed by thorough washing in PBS and incubation with secondary antibodies: for claudin-1, claudin-12, and claudin-19, Alexa fluor 555 donkey anti-rabbit antibodies (1:1000, Life Technologies, Invitrogen, MolecularProbes Inc., Eugene OR, USA, A31572) were used. The anti-claudin-5 and -ZO-1 antibodies were already conjugated with Alexa fluor 488. Before mounting, the samples were washed, and Hoechst 33342 solution was applied for five minutes at room temperature for counterstaining the nuclei (1 µg/mL in PBS, Thermo Scientific, Waltham, MA, USA). The sections were mounted with Vectashield Antifade Mounting Medium (Vector Laboratories, Burlingame, CA, USA).

Imaging of the fluorophore labeled sections was performed within one session with the same settings for each antibody (Biorevo BZ-9000-E, Keyence, Osaka, Japan). All images were saved as RGB 8-bit Tagged Image File Format (TIFF) files for further analysis. 

### 4.6. Laser Microdissection

**Figure 9:**
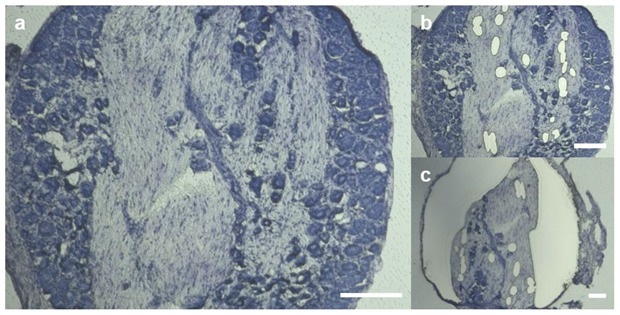
Stepwise sample acquisition of specific rat DRG regions via laser microdissection. Area specific samples of sham operated and rats 1 d after CCI were prepared after toluidine staining. Images of representative sections before dissection (**a**), and after acquisition of vessels in the FRR (**b**) and removal of the NRR (**c**) are shown. Scale bars measure 200 µm.

DRGs from Wistar rats were embedded in Tissue-Tek O.C.T compound (Sakura Finetek Europe B.V., AV Alphen aan den Rijn, The Netherlands) and stored at −80 °C. Cryosections of 20 µm were collected on Arcturus^®^ polyethylene naphtalate membrane slides (Applied Biosystems, Foster City, CA, United States) using a cryostat (Leica Biosystems CM3050 S Research Cryostat, Leica Biosystems Nussloch GmbH, Nussloch, Germany). The slides were treated with Rnase AWAY^®^ spray (Sigma Aldrich, St. Louis, MO, USA) before section collection. Before laser microdissection (LMD), sections were stained with toluidine blue. The slides were examined under a microscope coupled with a 355 nm laser (Leica^®^). Using the Leica LMD V7.6 software. capillaries were delimited and cut in the fiber rich area. Then, the entire neuron rich area was selected and sectioned as demonstrated in Figure 9. Samples were collected by gravity in 0.2 mL PCR SoftTubes (Biozym Scientific GmbH, Hessisch Oldendorf, Niedersachsen, Germany) and stored at −80 °C until reverse transcription qPCR.

### 4.7. Permeability of DRG Capillaries

Anaesthetized rats were laid down in a supine position on a pad. The 5th intercostal space was opened and enlarged by a retractor to open the thorax. The pericardium was stripped, exposing the heart anterior wall, and 12 mL of FITC dextran (70 kDa; 10 mg/mL; Sigma-Aldrich, St. Loui, MO, USA, FD70) or Hoechst 33342 (562 Da; 10 mg/mL; Sigma-Aldrich, St. Louis, MO, USA, 14533) solution was injected into the left ventricle using a syringe. The dye-perfused rats were sacrificed by decapitation after 1 min. DRGs were dissected and embedded in Tissue-Tek. Frozen samples were cut into 10 µm-thick sections on a cryostat at −20 °C. Without any fixation, microscope glass slides containing tissue sections were mounted and were imaged by fluorescence microscopy. The permeability of the DRG was determined by the measuring the fluorescence of FITC dextran in the NRR and FRR.

### 4.8. Image Analysis

All acquired images were analyzed with Fiji/ImageJ (version 1.52e, Open Source). Brightfield images of rat DRGs were used to distinguish the fiber rich region (FRR) and the neuron rich region (NRR), and their putative epi-/perineuria (FRR-EPN, NRR-EPN), as demonstrated in Figure 1a; and they were saved as regions of interest (ROIs). For vessel specific analysis, ROIs were created using vWF counterstaining and brightfield images.

### 4.9. Statistical Analysis

RStudio (version 1.1447, Open Source) was used for statistical analysis and plot generation. Datasets were tested for normal distribution and variance homogeneity using the Shapiro–Wilk normality test and Levene’s test of equality of variances. Datapoints were distributed normally with homogenous variances if not stated otherwise. Statistical significance of paw withdrawal threshold (Figure 2) was tested via repeated measures ANOVA. mRNA data was tested using Welch’s two sample *t*-test or Wilcoxon rank sum test if criteria for the *t*-test were not met. Two-factor ANOVA followed by Tukey’s test was used for analysis of immunoreactivity studies. All plots show means ± standard deviations. Significance was assumed as *p* < 0.05 (* *p* < 0.05; ** *p* < 0.01; *** *p* < 0.001) [55].

## 5. Conclusions 

Neurons and non-neuronal cells in the DRG regulate the perceptual quality of pain. We found reduced claudin-5 protein and mRNA expression in the NRR of the DRG following peripheral nerve injury by CCI. Measuring dye diffusion, we observed that the permeability in the NRR is higher compared to FRR in the DRG in naïve rats. Despite of reduced claudin-5, permeability to small and large molecules remained unchanged. Nerve injury-induced macrophage accumulation in the NRR was increased, indicating a possible role of tight junction proteins in cell migration.

Further studies of the BDB will decipher its function and role in pain. Whether BDB permeability, e.g., in MR imaging, will be a suitable biomarker for painful affection of the PNS, has to be studied in the future in preclinical models and patient cohorts.

## Figures and Tables

**Table 1:** Regional expression of tight junction proteins within rat DRGs. Neuron-rich region (NRR), fiber-rich region (FRR), epi-/perineurium (EPN).

Protein	Expression Pattern
Claudin-1	NRR-EPN and FRR-EPN > NRR and FRR
Claudin-5 (in vessels)	FRR > NRR
ZO-1	FRR-EPN > NRR, FRR, NRR-EPN
Claudin-19	FRR > NRR, NRR-EPN, FRR-EPN

**Table 2:** List of primers used in qRT-PCR analysis.

Gene	Species	Forward Primers	Reverse Primers
***Gapdh***	rat	5′-AGTCTACTGGCGTCTTCAC-3′	5′-TCATATTTCTCGTGGTTCAC-3′
***Cldn1***	rat	5′-GGGACAACATCGTGACTGCT-3′	5′-CCACTAATGTCGCCAGACCTG-3′
***Cldn5***	rat	5′-AAATTCTGGGTCTGGTGCTG-3′	5′-GCCGGTCAAGGTAACAAAGA-3′
***Cldn12***	rat	5′-AACTGGCCAAGTGTCTGGTC-3′	5′-AGACCCCCTGAGCTAGCAAT-3′
***Cldn19***	rat	5′-TGCTGAAGGACCCATCTG-3′	5′-TGTGCTTGCTGTGAGAACTG-3′
***ZO-1 (Tjp1)***	rat	5′-CACGATGCTCAGAGACGAAGG-3′	5′-TTCTACATATGGAAGTTGGGGATC-3′

## Abbreviations

BDB	blood–DRG barrier
CCI	chronic constriction injury
cldn1	claudin-1
cldn5	claudin-5
cldn12	claudin-12
cldn19	claudin-19
DRG	dorsal root ganglion
EPN	epi-/perineurium
FRR	fiber-rich region
IR	immunoreactivity
LMD	laser microdissection
MB	myelin barrier
NRR	neuron-rich region
vWF	von Willebrandt factor
